# Zinc Finger-Homeodomain Transcriptional Factors (ZHDs) in Upland Cotton (*Gossypium hirsutum*): Genome-Wide Identification and Expression Analysis in Fiber Development

**DOI:** 10.3389/fgene.2018.00357

**Published:** 2018-10-09

**Authors:** Muhammad Abdullah, Xi Cheng, Yunpeng Cao, Xueqiang Su, Muhammad Aamir Manzoor, Junshan Gao, Yongping Cai, Yi Lin

**Affiliations:** School of Life Sciences, Anhui Agricultural University, Hefei, China

**Keywords:** genome-wide analysis, ZHD, fiber development, abiotic stress, qRT-PCR

## Abstract

Zinc finger-homeodomain (ZHD) genes encode a family of plant-specific transcription factors that not only participate in the regulation of plant growth and development but also play an important role in the response to abiotic stress. The ZHD gene family has been studied in several model plants, including *Solanum lycopersicum, Zea mays, Oryza sativa*, and *Arabidopsis thaliana*. However, a comprehensive study of the genes of the ZHD family and their roles in fiber development and pigmentation in upland cotton has not been completed. To address this gap, we selected a brown fiber cultivar for our study; brown color in cotton is one of the most desired colors in the textile industry. The natural colored fibers require less processing and little dying, thereby eliminating dye costs and chemical residues. Using bioinformatics approaches, we identified 37 *GhZHD* genes from *Gossypium hirsutum* and then divided these genes into seven groups based on their phylogeny. The *GhZHD* genes were mostly conserved in each subfamily with minor variations in motif distribution and gene structure. These genes were largely distributed on 19 of the 26 upland cotton chromosomes. Among the *Gossypium* genomes, the paralogs and orthologs of the *GhZHD* genes were identified and further characterized. Furthermore, among the paralogs, we observed that the ZHD family duplications in *Gossypium* genomes (*G. hirsutum, G. arboreum*, and *G. raimondii*) were probably derived from segmental duplication or genome-wide duplication (GWD) events. Through a combination of qRT-PCR and proanthocyanidins (PA) accumulation analyses in brown cotton fibers, we concluded that the candidate genes involved in early fiber development and fiber pigment synthesis include the following: *GhZHD29, GhZHD35, GhZHD30, GhZHD31, GhZHD11, GhZHD27, GhZHD18, GhZHD15, GhZHD16, GhZHD22, GhZHD6, GhZHD33, GhZHD13, GhZHD5*, and *GhZHD23.* This study delivers insights into the evolution of the *GhZHD* genes in brown cotton, serves as a valuable resource for further studies, and identifies the conditions necessary for improving the quality of brown cotton fiber.

## Introduction

Zinc finger-homeodomain (ZHD) transcription factors (TFs) are major regulators of the body plan specification of higher plants and are especially involved in plant development (fiber development) and stress responses (Wang et al., 2015; Khatun et al., 2017). The first homeobox genes were identified in the fruit fly; however, these genes have since been isolated in many organisms, including fungi, plants, nematodes, and humans (Nam and Nei, 2005; Bhattacharjee et al., 2015). TFs can activate or repress target genes by directly binding to gene motifs or elements. Many TF families have evolved unique DNA-binding domains that direct their binding activities. The HD is a well-characterized DNA-binding domain that is encoded by a conserved 60 amino acid sequence (Mukherjee et al., 2009; Wang et al., 2014).

In plant and animal genomes, homeobox genes are part of a large gene family. Based on the number, nature, and spacing pattern, these genes can be categorized into different groups. Initially, zinc finger genes were categorized into the following groups: KNOX, ZM-HOX, BELL, AT-HB8, HAT, and GAL2 (Bharathan et al., 1997; Bhattacharjee et al., 2015). Over time, homeobox genes in rice were classified into ten subclasses: HD-Zip I, HD-Zip II, HD-Zip III, HD-Zip IV, KNOX I, KNOX II, BLH, WOX, PHD, and ZF-HD. Consequently, another systematic study on homeobox genes was carried out in which the genes were categorized into 14 subclasses, including the addition of some new classes, such as DDT, NDX, PHD, SAWADEE, LD, and PINTOX (Mukherjee et al., 2009). While some zinc fingers (C_2_H_2_, C_2_C_2_, and C_3_H) interact with one zinc ion, new approaches demonstrated that the animal Lin-11/Is1-1/Mec-3 (LIM) domain and plant RING finger domains interact with two zinc ions (Halbach et al., 2000; Englbrecht et al., 2004; Yanagisawa, 2004; Wang et al., 2014).

The first cluster of novel ZHD proteins was isolated from *Flaveria* as a potential regulator of the gene encoding C4 phosphoenolpyruvate carboxylase (PEPCase) (Windhovel et al., 2001). Windhovel et al. (2001) reported that the ZHD domain is capable of binding DNA, predominantly to the regulatory region of the C_4_ PEPCase genes. Subsequently, Windhovel et al. (2001) also described that ZF domains are not only intricately involved in DNA binding but also boost the protein–DNA interactions facilitated by the HD domain. A large number of studies on ZHD family genes have been performed in various plants, including *Arabidopsis thaliana* (Tan and Irish, 2006), *Glycine max* (Deng et al., 2002), *Oryza sativa* (Jørgensen et al., 1999), and *Triticum aestivum* (Bhattacharjee and Jain, 2013). Many members of the ZHD class are critical components in the regulation of blue light signaling, vascular development, biogenesis of the outer cell layer of plant organs, and the response to stress, in addition to controlling anthocyanin processes. While ZHD proteins were first reported to have a potential role in the regulation of floral development, it was later found that an Arabidopsis ZHD protein (*AtZHD1*) could bind to the promoter of *EARLY RESPONSE TO DEHYDRATION STRESS 1* (*ERD1*). For example, the expression pattern of *AtZHD1* is induced by abscisic acid, salt stress, and dehydration (Tran et al., 2007; Wang et al., 2014). In addition, ZHD proteins can interact with some NAC proteins and the simultaneous overexpression of ZHD and NAC genes increased drought tolerance in Arabidopsis (Tran et al., 2007; Hu et al., 2008). To date, 14 ZHD genes in Arabidopsis have been identified and characterized. Recently, the functions of ZHD genes in some other crops have been reported. For example, four rice ZHD genes have also been associated with gene regulation. Additionally, two soybean proteins, *GmZHD1* and *GmZHD2*, have been found to bind to the promoter of the gene encoding calmodulin isoform 4 (GmCaM4) and increase its expression upon pathogen stimulation (Park et al., 2007; Hu et al., 2008; Wang et al., 2014). Hu et al. (2008) stated that MIF1 interacts with ZHD proteins and that the overexpression of MIF1 interfered with the normal functions of ZHD proteins. If this is true, ZHD proteins may play important roles in regulating plant physiology and development. However, while the function of ZHD genes has been elucidated in Arabidopsis and other model crops, the functions of these genes in fiber development in *Gossypium hirsutum* have not yet been identified. *G. hirsutum* is one of the most valuable agricultural crops in the world and has been extensively studied on the developmental and physiological levels. *G. hirsutum* is a heterologous tetraploid cotton containing AA and DD genomes, which were formed approximately 1–2 million years ago. It is widely believed that *G. arboreum* and *G. raimondii* were the donators of the A and D chromosomes, respectively (Paterson et al., 2012; Wang et al., 2012; Chen et al., 2017a,b). The availability of complete *Gossypium* genome sequences makes it possible to examine and identify transcriptomic differences, duplications, and family sizes on a genome-wide scale spanning a broad evolutionary distance in the plant kingdom. Here, we report the identification of ZHD genes using *Gossypium* genome sequences and describe their characteristics, including phylogenetic and syntenic analyses, gene duplications, chromosomal locations, evolutionary mechanism, PA content, and expression differences during various fiber development stages. Our results provide a valuable foundation for future studies on ZHD proteins in brown cotton to facilitate functional analysis.

## Materials and Methods

### Plant Material

The brown cotton plant line Zongcaixuan No. 1 was used in these experiments at the High Technology Agricultural Park of Anhui Agricultural University (Hefei, Anhui, China). In July 2017, 60 plants in the blooming stage with good growth characteristics were selected. The experimental material was frozen in liquid nitrogen and quickly transferred to the laboratory refrigerator. The RNA of cotton fibers at 6, 12, 18, 24, and 30 days post-anthesis (DPA) was isolated for this study.

### Genomic Resources for the Screening of *ZHD* Genes in *Gossypium* Genomes

To identify the *ZHD* genes in *Gossypium* genomes, the genome sequences of the cotton species *Gossypium arboreum* (BJI, version 1.0), *G. raimondii* (JGI, version 2.0), and *G. hirsutum* (NAU, version 1.1) were downloaded from COTTONGEN^1^ (Yu et al., 2014), according to previously reported methods (Cao et al., 2017; Su et al., 2017; Abdullah et al., 2018b). Proteins with ZHD domains (PF04770) were retrieved from the Pfam database. HMMER software, which uses the hidden Markov model (HMM), was used on the *Gossypium* sequences with an *e*-value cut off of 0.001. Subsequently, we verified all sequences using various tools (Pfam, InterProScan database, NCBI, and SMART databases) (Zdobnov and Apweiler, 2001; Bateman, 2002; Letunic et al., 2012; Finn et al., 2014). The ExPASy program^2^ was used to determine the molecular weight and isoelectric points of the identified proteins (Gasteiger et al., 2003).

### Phylogenetic and Gene Structure Analysis

CLUSTAL_X software was used to perform the alignments of all the ZHD amino acid sequences using default parameters (Thompson et al., 1997). The phylogenetic tree was generated with MEGA 5.1 software using full-length sequences by using the maximum likelihood (ML) method with 1,000 bootstrap replications. The map of exon–intron structures of the *GhZHD* genes was analyzed by the Gene Structure Display Server 2.0 (GSDS)^3^. The MEME online tool^4^ was used to search the conservative motifs of GhZHD proteins, with a maximum width of 200 amino acids, a limit of 20 motifs, and all other default parameters (Bailey et al., 2015). Additionally, Pfam, InterProScan, and SMART databases were used to annotate these motifs (Zdobnov and Apweiler, 2001; Bateman, 2002; Letunic et al., 2012).

### Interspecies Microsynteny and *Cis*-Acting Element Analysis

The Multiple Collinearity Scan toolkit (MCScanX package with default parameters) was used to determine microsynteny among the *Gossypium* genomes. The *ZHD* genes of *G. hirsutum, G. arboreum*, and *G. raimondii* were ordered according to their evolutionary tree classification. We examined the putative promoter sequence from each *GhZHD* coding sequence, which is defined as the 1,500 bp upstream of the start codon (TTS), and analyzed the *cis*-elements using the PLANT CARE program^5^.

### Calculation of Non-synonymous (Ka) to Synonymous (Ks) Substitution Rates

DnaSP v5.0 software was used to determine the synonymous (Ks) and non-synonymous (Ka) nucleotide substitution rates. Each duplicated gene pair’s Ka/Ks ratio was calculated to determine the selection pressure. Sliding window analyses were performed for each duplicated gene pair to analyze the synonymous and non-synonymous substitution rates of encoding site paralogs.

### Physical Localization and Expansion Patterns

Information on the specific location of all *GhZHD* genes was obtained from genome annotation data and the chromosomal location of each gene was mapped using MapInspect software^6^. The expansion patterns of *GhZHD* genes in *G. hirsutum* were examined using MCScanX software with default parameters (Wang et al., 2015).

### Expression Analysis

The RNA-Seq data derived from the TM-1 transcriptome of the Cotton Functional Genomics Database^7^ (Zhu et al., 2017) were used to analyze the *GhZHD* gene expression profiles in *G. hirsutum*. In terms of expression level, we considered a gene expressed if its log2 (FPKM) value was higher than 1, and not expressed if its log2 (FPKM) value was equal to or less than 1. *G. hirsutum GhZHD* gene expression profiles were visualized using R software.

### RNA Extraction and Quantitative Real-Time PCR (qRT-PCR) Analysis

Total RNA was extracted and reverse-transcribed from brown cotton fiber at 6, 12, 18, 24, and 30 DPA using the Tiangen plant RNA extraction kit (Beijing, China). We designed specific primers (**Supplementary Table S3**) using Primer Premier 6 software. All primers are listed in **Supplementary Table S3**. The qRT-PCR analysis was performed using SYBR Green Master Mix (Takara, Japan) and detected with a CFX96 Touch^TM^ Real-time PCR Detection System (Singapore). Relative expression levels were calculated using the 2^-ΔΔCt^ method (Livak and Schmittgen, 2001).

### Proanthocyanidins (PA) Content

According to previously described methods (Ikegami et al., 2009), brown cotton fiber bolls at 6, 12, 18, 24, and 30 DPA were stripped, extracted with 80% methanol and placed into ultrasonic extraction for 30 min. Next, the samples were centrifuged for 15 min and the resulting supernatant was analyzed for soluble PAs. A methanol solution containing 1% HCl was added to the precipitate and the solution was placed in a 6°C water bath for 1 h. After centrifugation for 15 min, the supernatants contained the insoluble PAs and the PA content was determined by spectrophotometry according to a standard curve of catechins, which were used as controls (Li et al., 1996). For each experiment, three biological replicates were performed.

## Results

### Identification of *ZHD* Genes in *Gossypium* Genomes

To identify potential *ZHD* domain-encoding genes of *G. hirsutum, G. arboreum*, and *G. raimondii*, we obtained the ZHD domain (PF04770) from the Pfam database and generated an HMM profile with the HMMER 3.0 package. After removing repetitive sequences and any sequence lacking the ZHD domain, we obtained a total of 37 non-redundant *GhZHD* genes. The presence of 37 *ZHD* genes in *G. hirsutum* is greater than several in other species, including *G. arboreum* (22 *ZHD* genes), *G. raimondii* (19 *ZHD* genes), Chinese cabbage (31 *ZHD* genes) (Wang et al., 2015), grapes (13 *ZHD* genes) (Wang et al., 2014), and tomato (22 *ZHD* genes) (Khatun et al., 2017; **Supplementary Table S1**). We designated the genes *GhZHD1*–*GhZHD37* according to their order on the chromosomes (**Supplementary Figure S1**). The lengths (aa) of the proteins encoded by all of the *GhZHD* family members varied from 396 to 1944 aa, with an average length of 818 aa. Similarly, the molecular weight (MW) and the isoelectronic point (IP) varied from 36646.47 to 15391.95 kDa and from 9.12 to 6.54, respectively. The details of all 37 *GhZHD* genes, including physicochemical characteristics such as chromosome location, gene identifier, protein length (aa), molecular weight (MW), and isoelectric point (pI) are reported in **Supplementary Table S1**.

### Phylogenetic and Gene Structure Analysis of ZHD Genes

**FIGURE 1:**
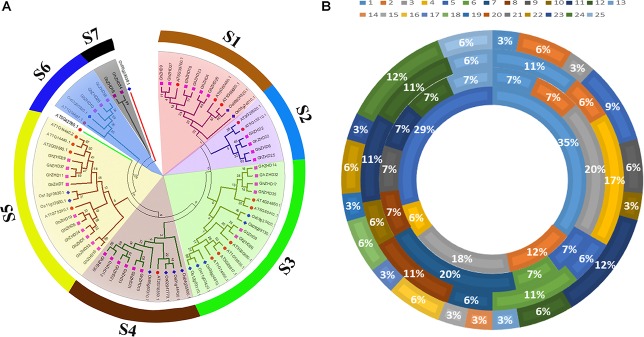
Phylogenetic tree of ZHD proteins from upland cotton, rice, maize, and Arabidopsis. The tree was generated with MEGA 5 software using the neighbor-joining method **(A)**. The chromosomal distribution and percentage of ZHD family genes shared in *G. hirsutum, G. arboreum, G. raimondii*, and Arabidopsis. The outermost circle represents the chromosomes of *G. hirsutum*, followed by *G. arboreum* and *G. raimondii*, with the innermost circle representing Arabidopsis **(B)**.

To gain insight into the evolutionary relationships of the upland cotton GhZHD gene family, a phylogenetic analysis was conducted from the 70 amino acid sequences of *G. hirsutum*, Arabidopsis (*A. thaliana*), maize (*Zea mays*), and rice (*O. sativa*) (**Figure 1A**). The phylogenetic tree was drawn based on sequence similarity and topology using Mega 5.1 software with the ML method and 1,000 bootstrap replications. After an examination of this phylogenetic tree, the *GhZHD* genes could be categorized into seven well-conserved clades (s1–s7) with bootstrap support. Clade s5 was the largest, containing 16 *GhZHD* members (approximately 22% of the total *GhZHD* genes), while clade s7 was the smallest, containing only two members. Numerous *G. hirsutum GhZHD* genes were not clustered with Arabidopsis *ZHD* genes. These *GhZHD* genes may have developed in upland cotton after diverging from the last common ancestor, or they may have been lost in Arabidopsis.

To further evaluate the diversity of upland cotton ZHD proteins, we used the MEME online program to predict conserved protein motifs. Five conserved motifs were identified in each comparison and labeled as Motif 1 through Motif 5 (**Supplementary Figure S2**). Interestingly, fewer motifs were observed in the *GhZHD* genes of upland cotton compared to other crops, including Arabidopsis, tomato, and Chinese cabbage (Wang et al., 2014; Khatun et al., 2017). Motif 1 and Motif 2 were the most common motifs and comprised the ZHD dimer domain. Most of the *GhZHDs* contained Motif 1 and Motif 2, suggesting that *GhZHD* genes have a conserved ZHD domain.

In addition to identifying conserved protein motifs, we also analyzed the structural diversity of upland cotton *GhZHD* genes. As shown in **Supplementary Figure S2**, most members of the same group have a similar exon–intron structure. We also highlighted domain positions (red color) within the exon–intron structure. Interestingly, after comparing the cDNA and genomic sequences of *GhZHD* genes, we observed that most of the *GhZHD* genes have only one exon and no introns. This unique pattern of *GhZHD* genes is different from other TFs. This intronless feature of this gene family indicates that *GhZHD* genes are less likely to undergo alternative splicing. This also indicates that the GhZHD gene family has a relatively fixed function compared to other TFs. Additionally, this feature significantly facilitates the annotation and identification of ZHD homologs in current and newly sequenced genomes.

### Chromosomal Localization and Microsynteny Analysis

To determine the chromosomal localization of *GhZHD* genes in the genome of upland cotton, chromosome maps were constructed based on genome annotation, with the exception of three genes that were located on the scaffold (**Supplementary Figure S1**). We observed that the 37 *GhZHD* genes were spread across 20 chromosomes with a non-random distribution. Only one *GhZHD* gene each was located on chromosomes 1, 3, 10, 13, 14, 15, 17, 19, and 23, while the 11th and 24th chromosomes contained a maximum number of four *GhZHD* genes each (12%). We also illustrated the percentage of *ZHD* genes in *G. hirsutum, G. arboreum, G. arboreum*, and Arabidopsis (**Figure 1B**). Furthermore, relatively high densities of *GhZHD* genes were located at specific positions of some chromosomes, such as the bottom of Dt/chromosome 9 and the top of At/chromosome 5 (**Supplementary Figure S1**). We analyzed the ratio of non-synonymous substitutions (Ka) to synonymous substitutions (Ks) in the orthologous gene pairs (**Supplementary Table S2**) and found that most of the orthologous gene pairs had a Ka/Ks ratio of less than 1. According to the neutral theory, suggesting that their undergone purifying selection and the function was not clearly differentiated. In addition, a sliding window analysis was performed to determine the Ka/Ks ratios of the CDS sequences at different sites. This analysis demonstrated that the Ka/Ks ratios of some coding sites were greater than 1, suggesting that *GhZHD* genes have also undergone positive selection at some coding sites (**Supplementary Figure S3**).

**FIGURE 2:**
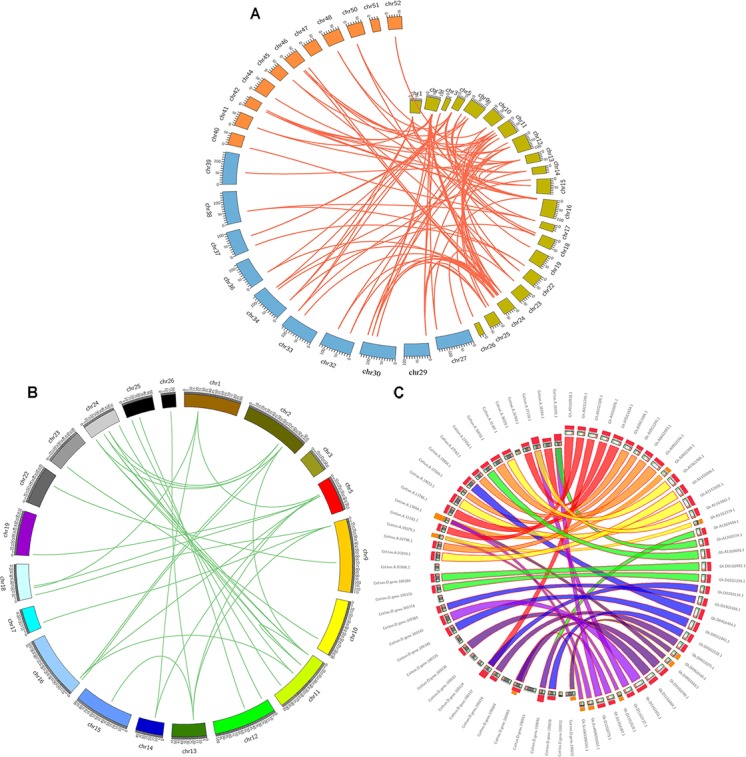
Microsynteny regions of *ZHD* genes among *Gossypium* genomes. **(A)** Synteny analyses of ZHD family genes between *G. hirsutum, G. arboreum*, and *G. raimondii.* The chromosome numbers of all three species are specified by different colors: olive green, blue, and orange represent the *G. hirsutum, G. arboreum*, and *G. raimondii* chromosomes, respectively. **(B)** Synteny of *ZHD* genes in the *Gossypium hirsutum* genome. The chromosome number is indicated on the outside by different colored boxes with the chromosome sequence lengths in megabases. Gene pairs with syntenic relationships are linked by a line. **(C)** Circles represent the *ZHD* gene sequence similarity among *Gossypium* genomes.

Genomic comparisons are a comparatively quick and active way to transfer genomic knowledge acquired in one taxon to another (Lyons et al., 2008). In this study, synteny analysis was carried out using MCScanX software and whole genome sequences to visualize the locations of homologous or orthologous genes (Cao et al., 2016; Cheng et al., 2017). The identification of orthologous *GhZHD* genes will further define the evolutionary history of this gene family. Microsynteny analysis was performed across the *G. hirsutum, G. arboreum*, and *G. raimondii* genomes. Among *G. hirsutum* and *G. raimondii*, 32 collinear blocks were identified, while 45 orthologous gene pairs were found between *G. hirsutum* and *G. arboreum* (**Figure 2A**). These results suggest that a closer relationship exists between *G. hirsutum* and *G. arboreum* than between *G. hirsutum* and *G. raimondii.* Additionally, 39 collinear blocks were identified in *G. hirsutum* (**Figures 2B,C**). A total of 72 collinear gene pairs were identified between the cotton genomes due to an ancient tetraploid process. Four *GhZHD* genes had no collinear block, suggesting that, in addition to the whole genome duplication event, an independent duplication event also occurred during the evolution of these species.

### Analysis of *Cis*-Elements in *ZHD* Genes

**FIGURE 3:**
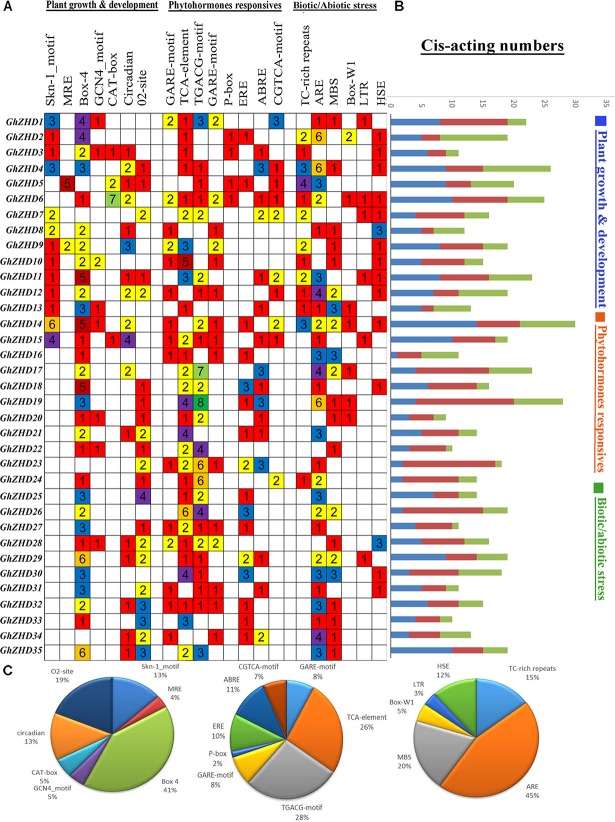
Identification of *cis*-acting elements in all *GhZHD* genes of *G. hirsutum*. **(A)** The different colors and numbers on the grid indicate the number of different promoter elements in each *GhZHD* gene. **(B)** The differently colored histograms represent the sum of *cis*-acting elements in each category. **(C)** Pie charts of different sizes indicate the ratio of each promoter element in each category.

Cis-elements in the promoter regions of genes provide cues for determining the stress-responsive or tissue-specific expression patterns in different environmental conditions. Significant positive correlations have been reported between multistimulus response genes and the density of *cis*-elements in their upstream regions (Tran et al., 2007; Walther et al., 2007). The PlantCARE database was used to identify potential stress- and hormone-responsive *cis*-acting elements in the promoter regions of *GhZHD* genes. The promoter regions, consisting of the genomic DNA sequences 1,500 bp upstream of the transcriptional start site (TTS), were examined for 37 *GhZHD* family genes. We detected a large number of *cis*-elements in the promoter regions of *GhZHD* genes (**Figure 3**). Our results suggest that GhZHD family genes may have different functions due to different types of *cis*-acting elements in their promoter regions. The *cis*-acting elements identified in our study can be classified into three categories: plant growth and development, phytohormone response, and biotic/abiotic stress response (Abdullah et al., 2018a). In the growth and development category, *cis*-acting elements were placed widely throughout the promoter regions, including Box 4 and MRE (responsible for plant growth in response to light), CAT-box (involved in meristem expression), circadian (required for circadian control), O2-site (involved in the regulation of zein metabolism), and Skn-1-motif and GCN4-motif (critical for endosperm expression). Box-4 covered the largest portion (41%) of the first category of *cis*-acting elements, followed by O2-site (19%), circadian (13%), and Skn-1-motif (13%) (**Figure 3**). In the phytohormone response category, we identified *cis*-acting elements including the P-box and GARE-motif (gibberellin-responsive elements), ERE (ethylene response), and ABRE (related to ABA). Remarkably, of the hormone responsive motifs, the TGACG *cis*-acting element (involved in auxin response) was the most common (28%), followed by the TCA-element (related to salicylic acid responsiveness) (26%). In the biotic/abiotic stress response category, a series of stress-related *cis*-acting elements were identified, including ARE (involved in anaerobic induction), HSE (heat stress), Box-W1 (participates in fungal elicitors), TC-rich repeats (general stress responses), and the GC-motif (involved in anoxia). We observed higher expression patterns for *GhZHD17, GhZHD22*, and *GhZHD26* in the RNA-Seq data and qRT-PCR analysis, and these particular genes also have a higher percentage of the *cis*-acting elements MBS (drought/salt stress responsiveness) and HSE (heat stress). Because the *GhZHD* genes contain MBS, HSE, and ABRE in their promoter regions, we propose that these genes are involved in salt, drought, and heat stress responses. These results indicate that *GhZHDs* have the potential to improve abiotic stress responses and may also respond to abiotic stresses (cold treatment, heat treatment, PEG-treatment, and salt treatment).

**FIGURE 4:**
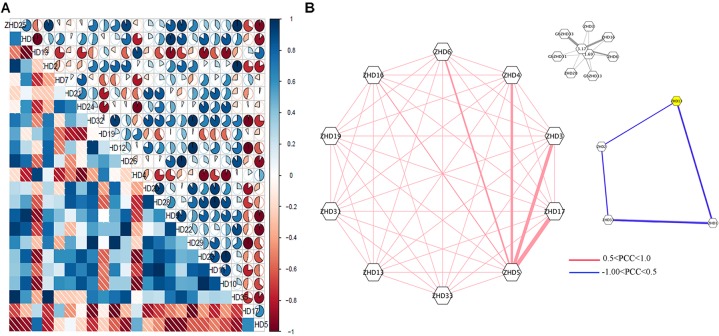
**(A)** Correlation analysis during abiotic stress. Correlation analysis was carried out using the R package program. Lower squares: correlations indicated by color and shading intensity. Upper circular symbols: each correlation is shown by the shades of blue and red and the size of the fan shape. Blue and red indicate a positive correlation and a negative correlation, respectively. **(B)** Co-regulatory networks. The co-regulatory networks of 37 *GhZHD* genes under stress treatments were established based on the Pearson correlation coefficients (PCCs) of these gene pairs using RNA-Seq data. The PCC of co-regulatory gene pairs was considered significant at the 0.05 significance level (*p*-value). Different color line styles indicate the different significance levels of the co-regulated gene pairs.

To obtain further insights into the roles of *GhZHD* genes during abiotic stresses, we analyzed their correlation networks based on the PCCs of their relative gene expression (**Figure 4**; Huang et al., 2015; Wang et al., 2017). Several genes showed positive or negative correlation with these treatments (cold, hot, and salt treated) at the various time points evaluated. There was a close relationship between some *GhZHD* genes, such as *GhZHD1* and *GhZHD13*. In addition, with the exception of *GhZHD5*, all of the other genes showed inverse correlations with *GhZHD17.* Surprisingly, we observed that duplicated *GhZHD* genes do not have closer relationships in terms of stress response compared to any other member of this gene family.

### *ZHD* Gene Expression in *Gossypium hirsutum*

Upland cotton is widely cultivated around the world. Previous studies have shown that biotic and abiotic stresses adversely affect the normal growth and fiber quality of this crop (Dhandapani et al., 2015). To gain further insights into the expression patterns of *GhZHD* genes, we used the available RNA-Seq data for fiber development, abiotic/biotic stress, tissue and organ development, and developmental biology.

**FIGURE 5:**
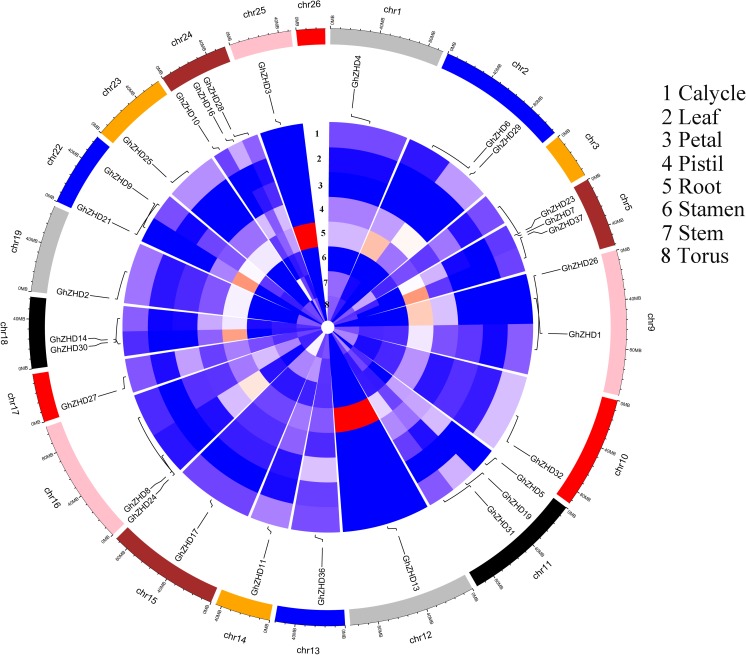
Circular heatmap (visualized using R software) depicting the stage-specific expression profiles of *GhZHD* genes. The FPKMs were calculated for expression values from RNA-Seq data.

**FIGURE 6:**
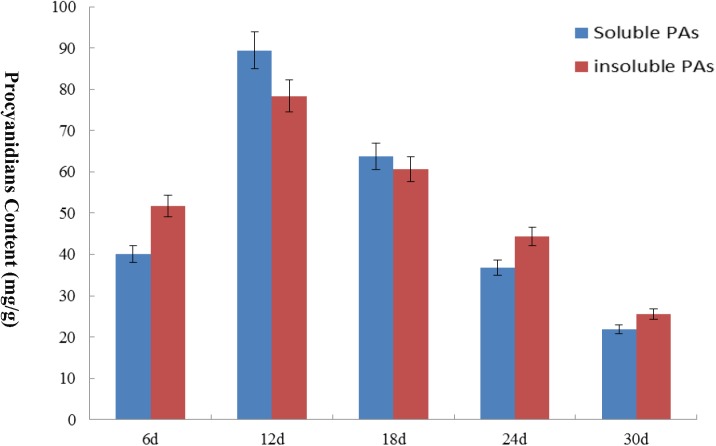
The PA content of brown cotton at different fiber development stages. The content levels of soluble PA and insoluble PA are expressed in different colors. The x-axis indicates different days of post-anthesis of cotton fibers, and the y-axis indicates the PA content. Error bars indicate SE.

**FIGURE 7:**
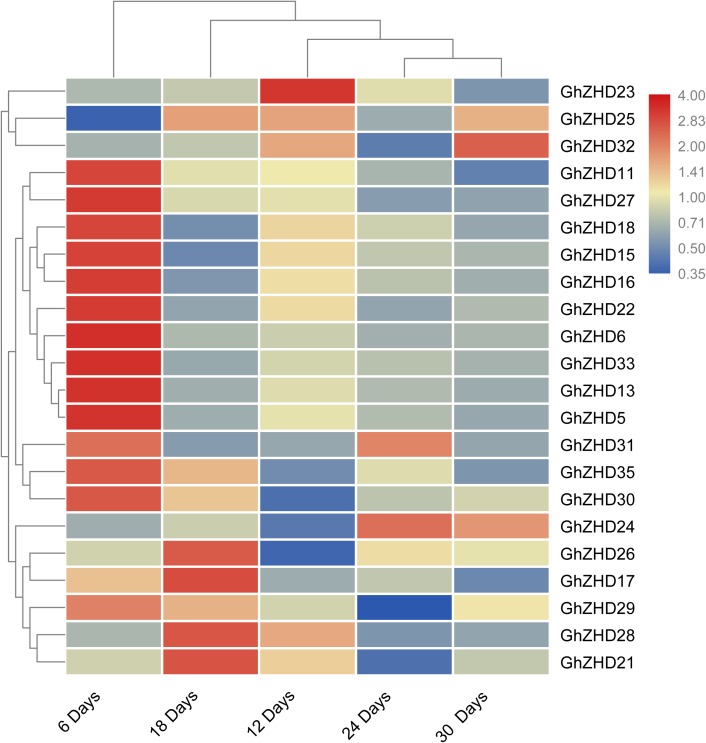
Expression patterns of *GhZHD* genes at different fiber developmental stages of brown cotton obtained by performing qRT-PCR. The relative expression levels were calculated using the 2^-ΔΔCt^ method.

We analyzed the transcript levels of the 37 *GhZHD* genes from eight upland cotton tissues (calycle, leaf, petal, pistil, root, stamen, stem, and torus). The expression levels of these genes are presented in a circle heatmap (**Figure 5**). Of the 37 *GhZHD* genes in upland cotton, the transcript levels (FPKM values) of seven genes indicated that they were not expressed in any tissue, while the remaining 30 were expressed in at least one tissue. Among them, 10 *GhZHD* genes were differentially expressed in all the examined tissues. Some genes showed tissue-specific expression, for example, *GhZHD6* was only expressed in the pistil. However, some genes were highly expressed in all of the examined tissues, including *GhZHD9, GhZHD22*, and *GhZHD4*. A large number of *GhZHD* genes showed preferential expression patterns in various tissues including the root (29), leaf (22), petal (16), pistil (32), stamen (20), stem (13), and torus (26). These results suggest that *GhZHD* genes may be involved in mediating plant growth and development. We also analyzed the RNA-Seq data collected during upland cotton fiber development. The transcript levels (FPKM values) of six genes indicated that they were not expressed in any fiber development stage, while the remaining genes were expressed in at least one fiber development stage. Based on this analysis, we selected candidate genes involved in fiber development. In brown cotton fibers, PAs are the key signs of pigment; hence, we examined whether such genes existed in brown cotton. We measured the PA content at different developmental stages of brown cotton fiber (**Figure 6**). Our results demonstrated that PA content gradually increased with the development of fiber and reached the highest level at 12 DPA, and then gradually decreased over time. Our results were consistent with previously published data on PA content during fiber development (Li et al., 2012; Feng et al., 2014). To identify the roles of *GhZHD* genes in PA accumulation during brown cotton fiber development, we designed primers based on the *GhZHD* gene sequences and performed qRT-PCR on 6, 12, 24, and 30 DPA brown cotton fiber (**Figure 7**). Interestingly, *GhZHD29, GhZHD35, GhZHD30, GhZHD31, GhZHD11, GhZHD27, GhZHD18, GhZHD15, GhZHD16, GhZHD22, GhZHD6, GhZHD33, GhZHD13, GhZHD5*, and *GhZHD23* showed higher expression at the early stages of cotton fiber development and their transcript levels gradually decreased. This is similar to the accumulation of PAs in brown cotton fibers. Therefore, we hypothesize that some of these *GhZHD* genes may be involved in PA accumulation. The expression patterns we obtained for these candidate genes by qRT-PCR were similar to their expression patterns in the analyzed RNA-Seq data.

## Discussion

Upland cotton is an important economic crop that is cultivated worldwide. The ZHD gene family is involved in a variety of processes, including plant development and physiological processes, as well as resistance to biotic/abiotic stresses. The plant-specific *ZHD* genes encode a family of TFs found in major groups of land plants, including vascular and non-vascular plants, but not found in prokaryotes, chlorophyte green algae, or fungi. The *ZHD* family genes may have evolved from a common ancestor or after the divergence of land plants from single-celled algae (Hu et al., 2008). The ZHD domain-containing gene family has been identified in many species, including Arabidopsis, maize, and rice. However, a systematic analysis of ZHD genes in upland cotton is still lacking. In this study, we aimed to complete a genome-wide survey of ZHD genes and their expression during fiber developmental processes and/or stress responses.

The number of *GhZHD* genes in *Gossypium* genomes was higher than in rice (15), Arabidopsis (17), *Brassica rapa* (31), or tomato (22). However, while the genome size of *G. hirsutum* (613 Mb) is smaller than that of tomato (950 Mb), it is larger than that of rice (441 Mb), Arabidopsis (164 Mb), and *Brassica rapa* (283.8 Mb). The number of ZHD family genes is relatively high in upland cotton, signifying that genome duplication events might have contributed to the expansion of *GhZHD* genes in this species. The identified protein characteristics and the conserved ZHD dimer domain of *GhZHD* family genes are consistent with those of other plant species, suggesting that the GhZHD proteins are structurally similar. The 37 GhZHD proteins were categorized into seven clades (s1–s7). Furthermore, we observed that most of the *GhZHD* genes were not closely related to the *ZHD* genes in Arabidopsis, consistent with the fact that upland cotton and Arabidopsis did not diverge from a recent common ancestor. The MEME server was used to identify the conserved motifs in the GhZHD proteins. Closely related members on the phylogenetic tree were found to have similar motifs, revealing the functional similarities between the proteins of the same subfamily. Intronless genes are very common in the genomes of higher eukaryotes (Louhichi et al., 2011). Gene structure analysis confirmed that all *GhZHD* genes of upland cotton are intronless (**Supplementary Figure S2**). Plant *ZHD* genes have previously been found to be intronless, and our data for upland cotton supports these findings (Wang et al., 2014, 2015; Khatun et al., 2017). Compared to other plants, the variable exon–intron structure of GhZHD family genes observed in upland cotton suggests that there is a structural divergence in the GhZHD gene family (**Supplementary Figure S2**). Furthermore, the similar exon–intron association among the different subfamilies suggests that these genes were highly conserved during evolution.

It is well known that gene duplication mechanisms (tandem/segmental duplication), transpositions, and whole genome duplications have a significant role in biological evolution (Xu et al., 2012). In our study, we observed that paralogous genes developed through segmental duplication, while no tandem duplications were observed for any gene pair, specifying that segmental duplication has played a significant role in the expansion of upland cotton ZHD family genes.

These plant-specific ZHD TFs are involved in various biological processes in plants including fiber development and responses to abiotic/biotic stress. The *GhZHD* genes contained specific DNA-binding motifs, such as the MYB motif, that are induced by several signals during stress conditions and various development processes (Yamaguchi-Shinozaki and Shinozaki, 2005). Recently, ZHD family proteins from Arabidopsis were shown to be induced by various stresses including salt and drought stresses (Nakashima and Yamaguchi-Shinozaki, 2006; Khatun et al., 2017). In our results, we observed that most *GhZHD* genes have preferential tissue expression patterns. Upland cotton fiber development is a complex biological process that ultimately leads to the production of crops for harvest. Fiber development is regulated by several transcriptional regulatory networks involving TFs. However, to date, the potential roles of GhZHD family genes in fiber development have not been characterized. Previous studies found that five key genes (CHI, F3H, DFR, ANS, and ANR) that participate in the PA synthesis pathway have higher expression levels in the early stages of fiber development (Xiao et al., 2007; Su et al., 2017; Chen et al., 2018), and after reaching their peak expression, they begin to gradually decrease. This result is consistent with the accumulation of PAs that we observed during the development of brown cotton fibers. Our results showed that the relative expression trends of *GhZHD29, GhZHD35, GhZHD30, GhZHD31, GhZHD11, GhZHD27, GhZHD18, GhZHD15, GhZHD16, GhZHD22, GhZHD6, GhZHD33, GhZHD13, GhZHD5*, and *GhZHD23* in brown cotton fiber were similar to the level of PA accumulation (**Figures 7**). Consequently, we proposed that one or more of these genes may affect the accumulation of PAs in cotton fiber. In our study, many stress-responsive and growth-regulatory *cis*-elements were widely distributed in the promoter regions of upland cotton *GhZHD* genes. Further studies on these putative *cis*-elements in the *GhZHD* genes of upland cotton are needed to unravel their complex regulatory mechanism.

## Conclusion

In summary, we conducted a genome-wide analysis of *GhZHD* genes in brown cotton, including gene structure analysis, chromosomal localization, conserved motif identification, phylogenetic relationship mapping, conserved microsynteny analysis, expression profiling during fiber development, PA contents, and functional divergence. The expression patterns of *GhZHD* genes during fiber development combined with PA synthesis analyses suggested that *GhZHD* genes may have functions in organ development and pigmentation. The identification and analysis of these *GhZHD* genes in brown cotton promotes a basic understanding of and provides a foundation for the extrapolation of the *GhZHD* gene function in future studies of brown cotton to improve fiber quality.

## Author Contributions

MA designed and performed the experiments. XC and YuC analyzed the data. XS, JG, and MAM contributed to reagents, materials, and analysis tools. MA wrote the paper. YoC and YL provided guidance on the whole manuscript. All authors reviewed and approved the final submission.

## Conflict of Interest Statement

The authors declare that the research was conducted in the absence of any commercial or financial relationships that could be construed as a potential conflict of interest.

## Footnotes

1 https://www.cottongen.org/

2 http://www.expasy.org/tools/

3 http://gsds.cbi.pku.edu.cn/

4 http://meme-suite.org/

5 http://bioinformatics.psb.ugent.be/webtools/plantcare/html/

6 http://mapinspect.software.informer.com/

7 https://cottonfgd.org/

## Supplementary Material

FIGURE S1 Chromosomal locations of the *GhZHD* genes in the genome of *Gossypium hirsutum*. The chromosome number is represented at the top of each chromosome and the left scale is in megabases (Mb).

FIGURE S2 Gene structure and distribution of the conserved motifs in the *ZHD* genes in upland cotton. **(A)** Untranslated region (UTR), introns, and exons are indicated by the blue box, thin line, and green box, respectively. **(B)** Conserved motifs located in each gene with their relative combined *p*-values.

FIGURE S3 Sliding window plots of candidate duplicated *ZHD* genes in upland cotton. The window size is 150 bp, and the step size is 9 bp. The x-axis indicates the synonymous distance within each gene.

TABLE S1 Detailed information about *GhZHD* genes from upland cotton.

TABLE S2 Synonymous and non-synonymous substitution rates for the duplication events in upland cotton.

TABLE S3 The primers used in qRT-PCR.
