# Supplementary figures and images for: Zinc Finger-Homeodomain Transcriptional Factors (ZHDs) in Upland Cotton (Gossypium hirsutum): Genome-Wide Identification and Expression Analysis in Fiber Development
Source: Front Genet. 2018 Oct 9;9:357. doi: 10.3389/fgene.2018.00357 (PMC6189526; doi:10.3389/fgene.2018.00357)

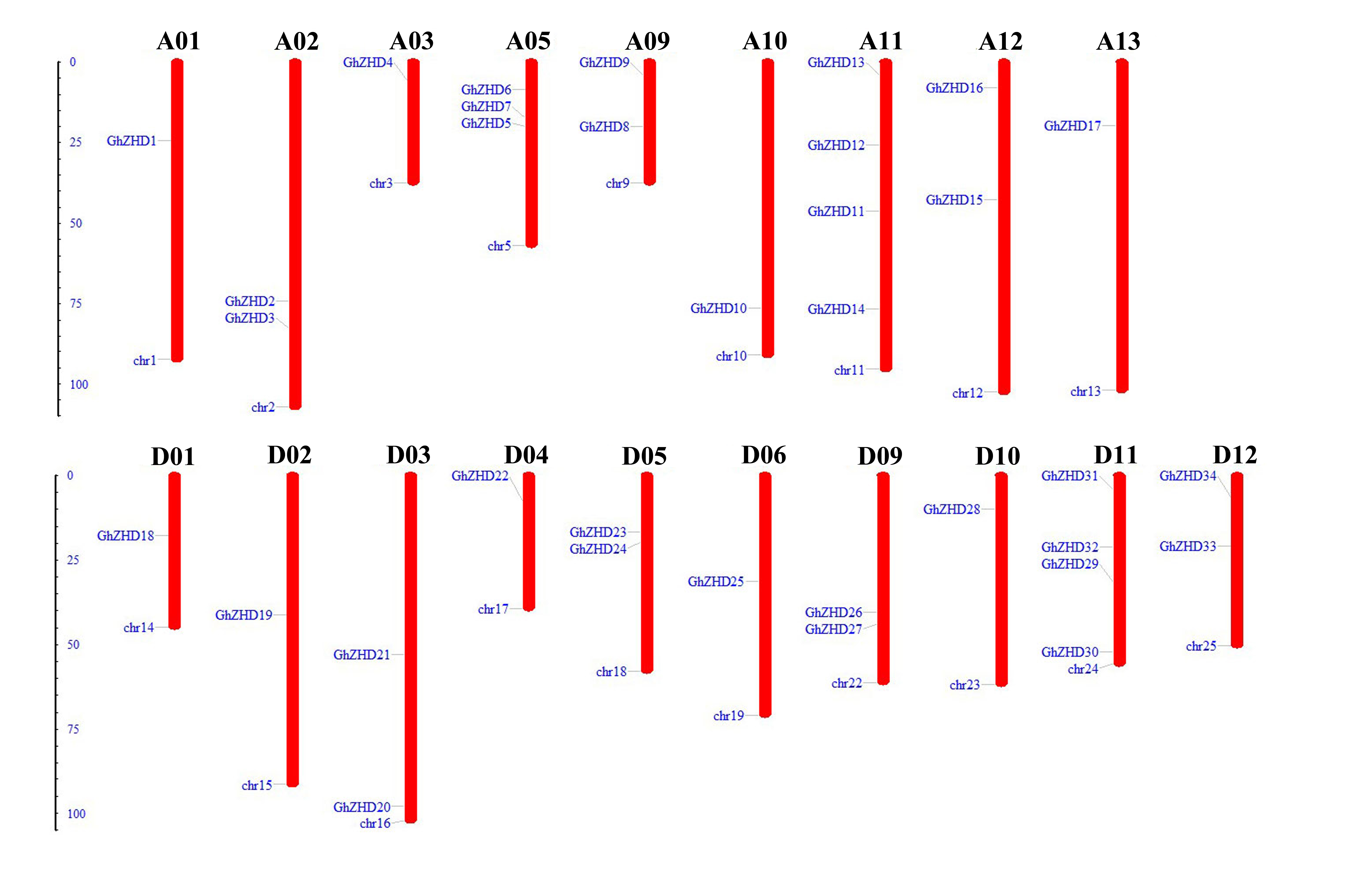

Supplement: FIGURE S1 — Chromosomal locations of the GhZHD genes in the genome of Gossypium hirsutum. The chromosome number is represented at the top of each chromosome and the left scale is in megabases (Mb). [file Image_1.JPEG]

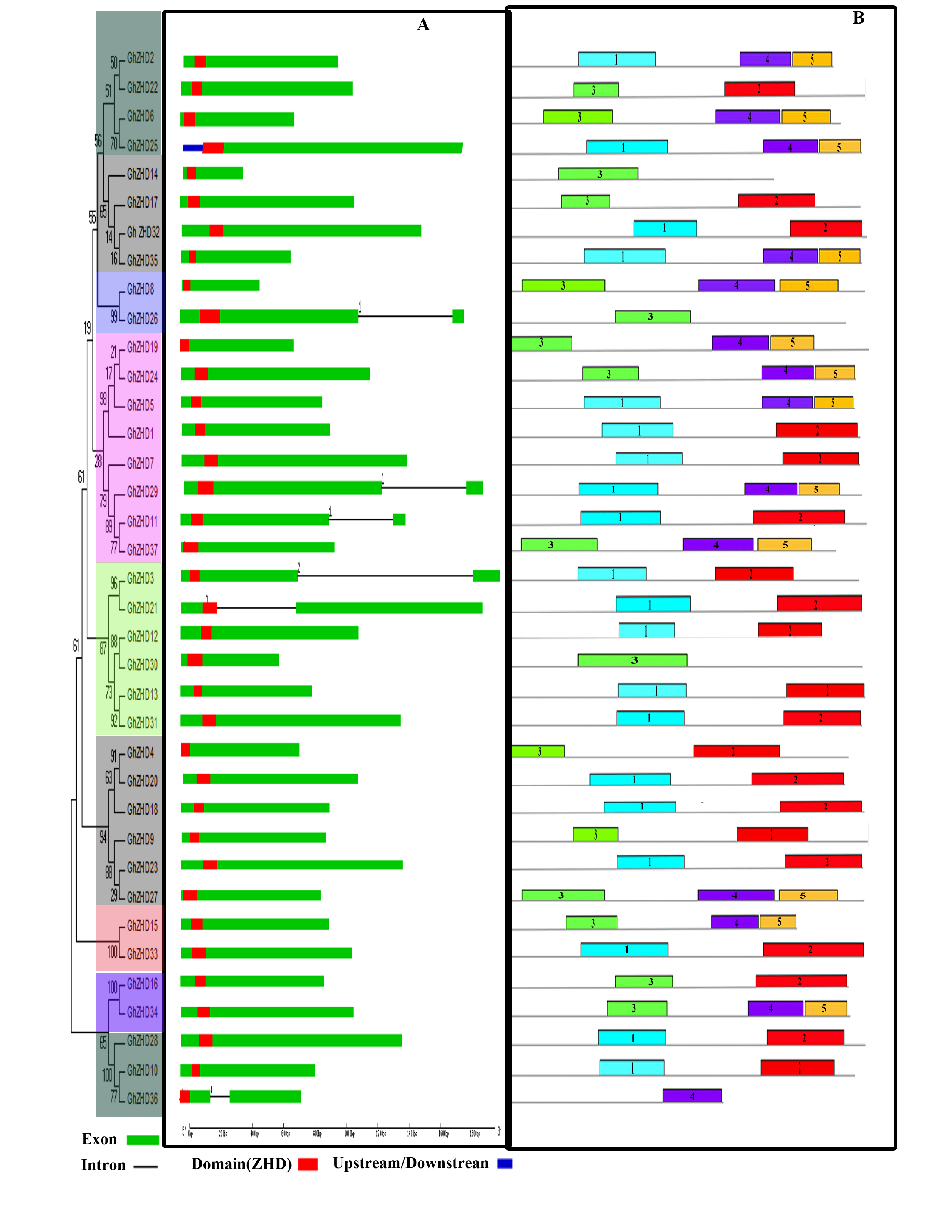

Supplement: FIGURE S2 — Gene structure and distribution of the conserved motifs in the ZHD genes in upland cotton. (A) Untranslated region (UTR), introns, and exons are indicated by the blue box, thin line, and green box, respectively. (B) Conserved motifs located in each gene with their relative combined p-values. [file Image_2.JPEG]

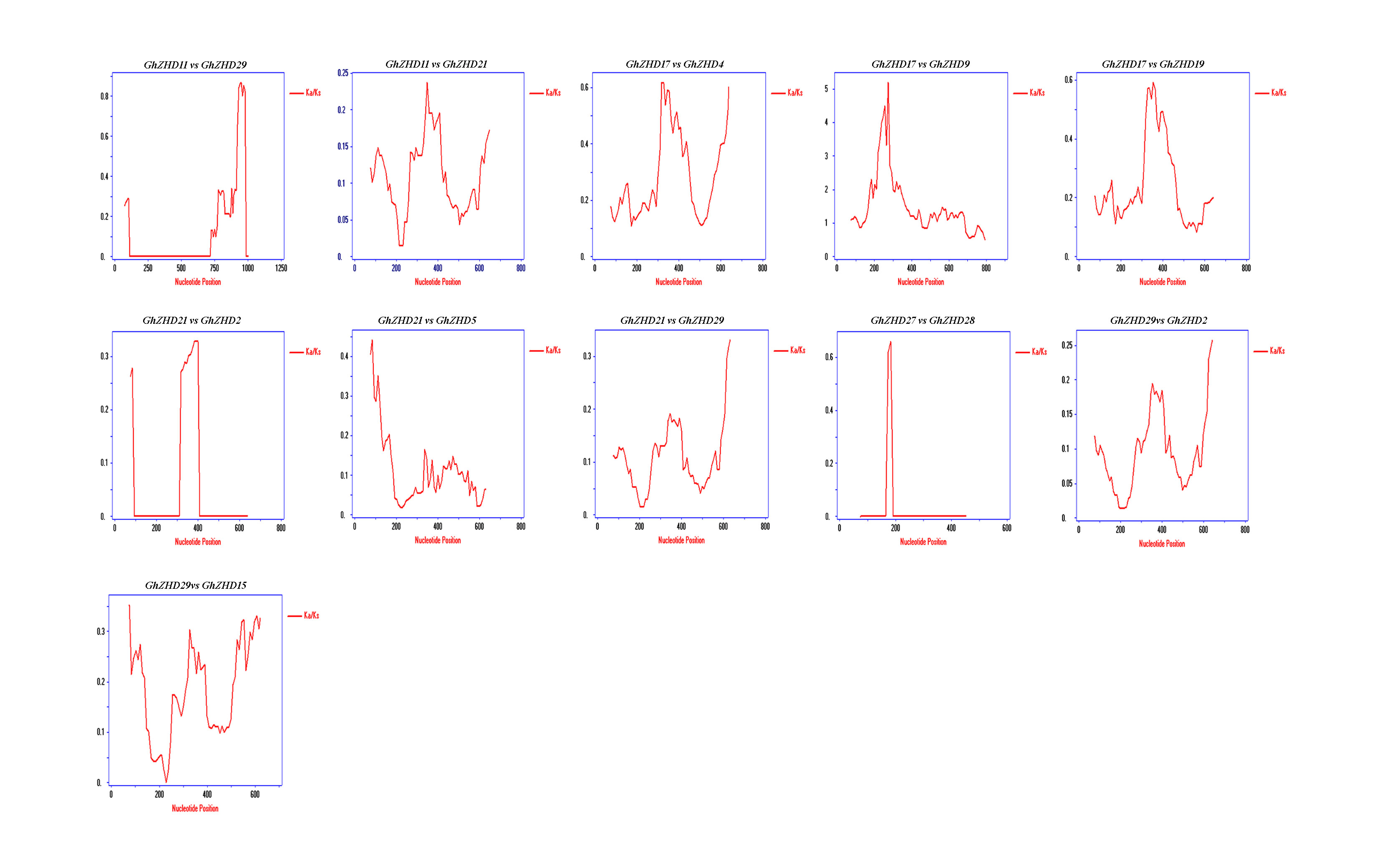

Supplement: FIGURE S3 — Sliding window plots of candidate duplicated ZHD genes in upland cotton. The window size is 150 bp, and the step size is 9 bp. The x-axis indicates the synonymous distance within each gene. [file Image_3.JPEG]
