# Supplementary material for: Zinc Finger-Homeodomain Transcriptional Factors (ZHDs) in Upland Cotton (Gossypium hirsutum): Genome-Wide Identification and Expression Analysis in Fiber Development
Source: Front Genet. 2018 Oct 9;9:357. doi: 10.3389/fgene.2018.00357 (PMC6189526; doi:10.3389/fgene.2018.00357)
Supplement: TABLE S1 — Detailed information about GhZHD genes from upland cotton. [file Table_1.DOCX]

| \| **Gene Name** \| **Gene ID** \| **Chromosome** \| **Start** \| **End** \| **Strand** \| **Length (bp)** \| **Molecular weight** \| **theoretical PI** \| \| --- \| --- \| --- \| --- \| --- \| --- \| --- \| --- \| --- \| \| GhZHD1 \| Gh_A01G0938 \| A01 \| 24,417,696 \| 24,418,541 \| - \| 846 \| 31898.51 \| 7.81 \| \| GhZHD2 \| Gh_A02G1249 \| A02 \| 74,019,988 \| 74,020,707 \| + \| 720 \| 27621.23 \| 7.43 \| \| GhZHD3 \| Gh_A02G1589 \| A02 \| 82,454,734 \| 82,456,677 \| + \| 1,944 \| 33147.71 \| 8.99 \| \| GhZHD4 \| Gh_A03G0876 \| A03 \| 55,849,141 \| 55,849,911 \| - \| 771 \| 29013.7 \| 7.85 \| \| GhZHD5 \| Gh_A05G1894 \| A05 \| 19,861,844 \| 19,862,617 \| + \| 774 \| 33301.95 \| 8.63 \| \| GhZHD6 \| Gh_A05G3240 \| A05 \| 84,762,810 \| 84,763,400 \| + \| 591 \| 28471.16 \| 7.38 \| \| GhZHD7 \| Gh_A05G1654 \| A05 \| 17,136,355 \| 17,137,254 \| - \| 900 \| 22292.79 \| 8.79 \| \| GhZHD8 \| Gh_A09G1569 \| A09 \| 69,018,176 \| 69,018,703 \| + \| 528 \| 27998.47 \| 6.54 \| \| GhZHD9 \| Gh_A09G0154 \| A09 \| 4,084,462 \| 4,085,442 \| + \| 981 \| 36342.67 \| 7.56 \| \| GhZHD10 \| Gh_A10G1400 \| A10 \| 76,228,093 \| 76,229,010 \| + \| 918 \| 20367.17 \| 8.43 \| \| GhZHD11 \| Gh_A11G1861 \| A11 \| 46,092,638 \| 46,093,710 \| + \| 1,073 \| 33835.64 \| 8.16 \| \| GhZHD12 \| Gh_A11G1692 \| A11 \| 25,809,865 \| 25,810,842 \| - \| 978 \| 33995.69 \| 9.11 \| \| GhZHD13 \| Gh_A11G0406 \| A11 \| 3,760,258 \| 3,761,151 \| - \| 894 \| 36646.47 \| 8.95 \| \| GhZHD14 \| Gh_A11G2219 \| A11 \| 76,563,232 \| 76,563,639 \| - \| 408 \| 29217.17 \| 6.59 \| \| GhZHD15 \| Gh_A12G0774 \| A12 \| 42,602,511 \| 42,603,206 \| + \| 696 \| 15996.66 \| 6.65 \| \| GhZHD16 \| Gh_A12G0404 \| A12 \| 7,900,756 \| 7,901,484 \| + \| 729 \| 28054.68 \| 9.12 \| \| GhZHD17 \| Gh_A13G0659 \| A13 \| 19,706,117 \| 19,707,004 \| - \| 888 \| 26517.16 \| 8.24 \| \| GhZHD18 \| Gh_D01G0982 \| D01 \| 17,625,720 \| 17,626,565 \| - \| 846 \| 32943.22 \| 8.18 \| \| GhZHD19 \| Gh_D02G1259 \| D02 \| 41,275,209 \| 41,275,979 \| - \| 771 \| 31932.53 \| 8.43 \| \| GhZHD20 \| Gh_D03G0134 \| D03 \| 980,804 \| 981,517 \| - \| 714 \| 28904.61 \| 8.24 \| \| GhZHD21 \| Gh_D03G0388 \| D03 \| 5,286,185 \| 5,287,346 \| + \| 1,162 \| 27217.57 \| 7.82 \| \| GhZHD22 \| Gh_D04G0444 \| D04 \| 7,194,128 \| 7,195,108 \| + \| 981 \| 30705.88 \| 7.1 \| \| GhZHD23 \| Gh_D05G1843 \| D05 \| 16,727,498 \| 16,728,397 \| - \| 900 \| 35972.19 \| 8.19 \| \| GhZHD24 \| Gh_D05G2128 \| D05 \| 19,923,948 \| 19,924,715 \| + \| 768 \| 33202.84 \| 8.44 \| \| GhZHD25 \| Gh_D06G0275 \| D06 \| 3,121,240 \| 3,121,980 \| + \| 741 \| 28143.67 \| 6.65 \| \| GhZHD26 \| Gh_D09G0146 \| D09 \| 4,051,015 \| 4,051,841 \| + \| 827 \| 27629.14 \| 6.44 \| \| GhZHD27 \| Gh_D09G1640 \| D09 \| 44,005,053 \| 44,005,580 \| + \| 528 \| 20588.51 \| 7.12 \| \| GhZHD28 \| Gh_D10G0799 \| D10 \| 9,795,079 \| 9,795,978 \| + \| 900 \| 20347.18 \| 8.43 \| \| GhZHD29 \| Gh_D11G2127 \| D11 \| 31,273,972 \| 31,275,038 \| - \| 1,067 \| 33243.07 \| 8.16 \| \| GhZHD30 \| Gh_D11G2528 \| D11 \| 52,168,845 \| 52,169,240 \| - \| 396 \| 33970.68 \| 8.97 \| \| GhZHD31 \| Gh_D11G0469 \| D11 \| 4,020,951 \| 4,021,844 \| - \| 894 \| 36348.07 \| 8.43 \| \| GhZHD32 \| Gh_D11G1851 \| D11 \| 21,238,006 \| 21,238,980 \| - \| 975 \| 29217.17 \| 6.59 \| \| GhZHD33 \| Gh_D12G0775 \| D12 \| 20,868,067 \| 20,868,762 \| + \| 696 \| 15391.95 \| 7.82 \| \| GhZHD34 \| Gh_D12G0397 \| D12 \| 6,343,474 \| 6,344,172 \| + \| 699 \| 26834.22 \| 8.94 \| \| GhZHD35 \| Gh_A06G1895 \| scaffold1263_A06 \| 114,508 \| 115,254 \| + \| 747 \| 26620.28 \| 8.52 \| \| GhZHD36 \| Gh_Sca069306G01 \| scaffold69306 \| 91 \| 582 \| + \| 492 \| 20367.17 \| 8.43 \| \| GhZHD37 \| Gh_Sca068050G01 \| scaffold68050 \| 8 \| 535 \| + \| 528 \| 15860.5 \| 6.25 \| |
| --- | --- | --- | --- | --- | --- | --- | --- | --- | --- | --- | --- | --- | --- | --- | --- | --- | --- | --- | --- | --- | --- | --- | --- | --- | --- | --- | --- | --- | --- | --- | --- | --- | --- | --- | --- | --- | --- | --- | --- | --- | --- | --- | --- | --- | --- | --- | --- | --- | --- | --- | --- | --- | --- | --- | --- | --- | --- | --- | --- | --- | --- | --- | --- | --- | --- | --- | --- | --- | --- | --- | --- | --- | --- | --- | --- | --- | --- | --- | --- | --- | --- | --- | --- | --- | --- | --- | --- | --- | --- | --- | --- | --- | --- | --- | --- | --- | --- | --- | --- | --- | --- | --- | --- | --- | --- | --- | --- | --- | --- | --- | --- | --- | --- | --- | --- | --- | --- | --- | --- | --- | --- | --- | --- | --- | --- | --- | --- | --- | --- | --- | --- | --- | --- | --- | --- | --- | --- | --- | --- | --- | --- | --- | --- | --- | --- | --- | --- | --- | --- | --- | --- | --- | --- | --- | --- | --- | --- | --- | --- | --- | --- | --- | --- | --- | --- | --- | --- | --- | --- | --- | --- | --- | --- | --- | --- | --- | --- | --- | --- | --- | --- | --- | --- | --- | --- | --- | --- | --- | --- | --- | --- | --- | --- | --- | --- | --- | --- | --- | --- | --- | --- | --- | --- | --- | --- | --- | --- | --- | --- | --- | --- | --- | --- | --- | --- | --- | --- | --- | --- | --- | --- | --- | --- | --- | --- | --- | --- | --- | --- | --- | --- | --- | --- | --- | --- | --- | --- | --- | --- | --- | --- | --- | --- | --- | --- | --- | --- | --- | --- | --- | --- | --- | --- | --- | --- | --- | --- | --- | --- | --- | --- | --- | --- | --- | --- | --- | --- | --- | --- | --- | --- | --- | --- | --- | --- | --- | --- | --- | --- | --- | --- | --- | --- | --- | --- | --- | --- | --- | --- | --- | --- | --- | --- | --- | --- | --- | --- | --- | --- | --- | --- | --- | --- | --- | --- | --- | --- | --- | --- | --- | --- | --- | --- | --- | --- | --- | --- | --- | --- | --- | --- | --- | --- | --- | --- | --- | --- | --- | --- | --- | --- | --- | --- | --- | --- | --- | --- | --- | --- | --- | --- | --- |

The detailed information of *ZHD* genes from *Gossypium.hirsutum*.
