# Supplementary material for: Zinc Finger-Homeodomain Transcriptional Factors (ZHDs) in Upland Cotton (Gossypium hirsutum): Genome-Wide Identification and Expression Analysis in Fiber Development
Source: Front Genet. 2018 Oct 9;9:357. doi: 10.3389/fgene.2018.00357 (PMC6189526; doi:10.3389/fgene.2018.00357)
Supplement: TABLE S2 — Synonymous and non-synonymous substitution rates for the duplication events in upland cotton. [file Table_2.DOCX]

| **Duplicate gene 1** | **Duplicate gene 2** | **ks** | **ka** | **ka/ks** | **selection** | **Duplication Type** |
| --- | --- | --- | --- | --- | --- | --- |
| ***GhZHD11*** | ***GhZHD29*** | 0.5114 | 0.0552 | 0.2833 | Purifying | Segmental |
| ***GhZHD11*** | ***GhZHD21*** | 0.0428 | 0.0108 | 0.0268 | Purifying | Segmental |
| ***GhZHD17*** | ***GhZHD4*** | 0.3888 | 0.1223 | 0.25555 | Purifying | Segmental |
| ***GhZHD17*** | ***GhZHD19*** | 0.4354 | 0.1149 | 0.27515 | Purifying | Segmental |
| ***GhZHD17*** | ***GhZHD9*** | 0.4941 | 0.5467 | 0.5204 | Purifying | Segmental |
| ***GhZHD21*** | ***GhZHD29*** | 0.4795 | 0.0505 | 0.265 | Purifying | Segmental |
| ***GhZHD21*** | ***GhZHD2*** | 0.0334 | 0.0036 | 0.0185 | Purifying | Segmental |
| ***GhZHD21*** | ***GhZHD5*** | 1.7195 | 0.2213 | 0.9704 | Purifying | Segmental |
| ***GhZHD27*** | ***GhZHD8*** | 0.0084 | 0.005 | 0.0067 | Purifying | Segmental |
| ***GhZHD29*** | ***GhZHD15*** | 0.3831 | 0.0768 | 0.22995 | Purifying | Segmental |
| ***GhZHD29*** | ***GhZHD2*** | 0.472 | 0.046 | 0.259 | Purifying | Segmental |
