# Supplementary material for: Zinc Finger-Homeodomain Transcriptional Factors (ZHDs) in Upland Cotton (Gossypium hirsutum): Genome-Wide Identification and Expression Analysis in Fiber Development
Source: Front Genet. 2018 Oct 9;9:357. doi: 10.3389/fgene.2018.00357 (PMC6189526; doi:10.3389/fgene.2018.00357)
Supplement: TABLE S3 — The primers used in qRT-PCR. [file Table_3.DOCX]

| **Gene ID** | **Forward** | **Reverse** |
| --- | --- | --- |
| ***GhZHD29*** | ACCATGAACCGTTCGATCTCTTCT | AGGCCAAGATAATAAGGAA |
| ***GhZHD35*** | TGTTCCACCAGGCTACGACTC | TGACCAAGAAGAAGAAATG |
| ***GhZHD30*** | TTCGTTCTCCTGGCTATGATCTCAA | CAGCAATTTCTTCGAACA |
| ***GhZHD31*** | ACTCTCATCTTCTTCTGCACCTATAA | CAAGCTGATTCTTCCTTAA |
| ***GhZHD11*** | TTCCTCTGCTGCTGTTGCTG | GAGCAAGAAGAGGAGATG |
| ***GhZHD27*** | ACCGCAATTTCCACCGCAAA | CTCTCTTTCTACCCTACAA |
| ***GhZHD18*** | AACCACAGGCAGCACACTTG | TGGAGAGTTCATGCCTAC |
| ***GhZHD15*** | CGCTTCCTCTGTTGCTGCTG | ACTCGCTAGTAAACTCAA |
| ***GhZHD16*** | TCAACATCTGATTCAGCCTCCACT | GCACTGCTGATTATCAAC |
| ***GhZHD22*** | CAAGCGTCACAAGCCTCACC | CCAAGCCTGTATCTTTCA |
| ***GhZHD6*** | ATTCCTTGTACCTCACCACCACTT | AAGCCAATCTCAACAAGC |
| ***GhZHD33*** | CGCTTCCTCTGTTGCTGCTG | ACTCGCTAGTAAACTCAA |
| ***GhZHD13*** | AGTCGTAATAATAAGCTGAACCAAGC | GCCAAGATAAGCAAATGG |
| ***GhZHD5*** | CCAGCAGGATACGACTCACTCT | AGACGAAGAAATGGGTATG |
| ***GhZHD21*** | CATCTCTTCTTCCTGCTCTTCCTGA | CCTGCTATCATCACTGTAG |
| ***GhZHD25*** | TGTTCCACCAGGCTACGATTCA | TGACCAAGAAGAAGAAATG |
| ***GhZHD28*** | CAACCATATCATACCTTCTTCAGCACC | GCACATGATCCATCATGA |
| ***GhZHD26*** | CCACCAACCTCACCACCACTA | GCCCAAATCTTTTACTAACG |
| ***GhZHD17*** | CCTCAAGAGCTGCACAATCCAC | CACGACCAAGTCTTTAAC |
| ***GhZHD23*** | CAGCCTTTGGTCTTCACTCGC | GAGGCCAAGAACATGATA |
| ***GhZHD24*** | ACTCACTCGGCAACTCGTCA | GACGAAGAAATGGGTATG |
| ***GhZHD32*** | ACCATGAACCGTTCGATCTCTTCT | AGGCCAAGATAATAAGGAA |
